# Decisional components of motor responses are not related to online response control: Evidence from lexical decision and speed-accuracy tradeoff manipulations

**DOI:** 10.3758/s13421-024-01619-3

**Published:** 2024-08-19

**Authors:** Michele Scaltritti, Elena Greatti, Simone Sulpizio

**Affiliations:** 1 Dipartimento Di Psicologia e Scienze Cognitive, Università Degli Studi Di Trento, Rovereto, Italy; 2 Dipartimento Di Neuroscienze Cognitive, Scuola Internazionale Di Studi Avanzati (SISSA), Trieste, Italy; 3 International School of Advanced Studies, Centro Di Neuroscienze, Università Di Camerino, Camerino, Italy; 4 Dipartimento Di Psicologia, Università Degli Studi Di Milano-Bicocca, Milan, Italy; 5 Milan Center for Neuroscience (NeuroMI), Università Degli Studi Di Milano-Bicocca, Milan, Italy

**Keywords:** Decision making, Speed-accuracy tradeoff, Lexical decision, Motor-response execution

## Abstract

Evidence suggests that decision processes can propagate to motor-response execution. However, the functional characterization of motor decisional components is not yet fully understood. By combining a classic lexical decision experiment with manipulations of speed-accuracy tradeoff (SAT), the present experiment assessed the hypothesis that decisional effects on chronometric measures of motor-response execution are related to online response control. The electromyographic (EMG) signal associated with manual button-press responses was used to dissociate the premotor component (from stimulus onset until the onset of the EMG activity) from the motor component (from EMG onset until the button-press), thus enabling the assessment of decision-related effects in terms of motor-response duration within single-trial reaction times. Other than replicating all the previously reported SAT effects, the experiment revealed hindered control processes when the instructions emphasized speed over accuracy, as indicated by measures of response control such as partial errors, fast errors, and correction likelihood. Nonetheless, the lexicality effect on motor responses, consisting of slower motor times for pseudowords compared to words, was impervious to any SAT modulation. The results suggest that SAT-induced variations in decision and response control policies may not be the prominent determinant of decision-related effects on motor times, highlighting the multiple “cognitive” components that affect peripheral response execution.

**Supplementary Information:**

The online version contains supplementary material available at 10.3758/s13421-024-01619-3.

## Introduction

Our actions, at least the simplest ones, may appear as the mere implementation of a commitment determined during prior decisional processes: first we decide, then we act. Insights from decision-making research reveal a more complex scenario. In the context of two-alternative choice experiments, hand movements within choice-reaching tasks (Song & Nakayama, 2009) and even button-press responses (Donner et al., 2009) unfold on par with the still-evolving decision processes, blurring the temporal and functional boundaries between decision and motor stages. Although empirical evidence is in agreement that the notion that motor-response execution still carries decisional contents, the functional characterization of the decisional component(s) putatively at play remains elusive.

A first hypothesis relates the “cognitive” effects observed at the motor level to the propagation of the same decisional variable that informs prior non-motor stages (Servant et al., 2021). This perspective is, however, challenged by cases of independence between effects observed across pre-motor and motor stages (Allain et al., 2004; Scaltritti et al., 2023a, 2023b; Weindel et al., 2021), which seem in contrast with the notion of a single decisional variable informing both levels of cognitive and motor processing. In a series of experiments exploiting lexical and object decision paradigms, previous evidence highlighted a selective propagation of decisional effects to the motor level (Scaltritti et al., 2023a, 2023b). Specifically, electromyographic (EMG) traces associated with button-press responses were exploited to separate, within single-trial reaction times (RTs), a premotor time (PMT), consisting of the interval between stimulus onset until the onset of the muscular activity, from the motor time (MT), capturing the time from the onset of EMG activity until the button-press (Botwinick & Thompson, 1966). When participants were instructed to classify letter strings as words or pseudowords (lexical decision), the slowdown in RTs for pseudowords (i.e., the lexicality effect) was reflected on both PMT and MT components. The same pattern surfaced in an object-decision task, featuring pictures of objects and pseudo-objects as stimuli. Differently, the effect of lexical frequency, that is the difference between frequent (e.g., *house*) and infrequent words (e.g., *sapphire*), was bounded to the PMT component, suggesting that not all the decision-related dynamics necessarily propagate from the premotor level to motor responses (for different perspectives on the dissociations between PMT and MT effects, see Dendauw et al., 2024; Servant et al., 2021).

An alternative hypothesis concerning the functional characterization of motor-decisional dynamics focuses on online processes of response control (Scaltritti et al., 2023a, 2023b). The literature on conflict tasks has highlighted the presence of online control processes acting on response activations (e.g., Burle et al., 2002; Spieser et al., 2015; Taylor et al., 2007) to overcome incorrect response tendencies and to detect and correct errors (Ramdani et al., 2013) during the unfolding of the motor responses. Similar response control mechanisms may be at play even in the context of the lexical decision task, particularly for pseudo-items, that is, for stimuli with no representation in long-term memory (Scaltritti et al., 2023a, 2023b). Within this perspective, one hypothesis is that the lexicality effect on MTs would reflect stronger response monitoring for pseudowords compared to words.

In lexical decision, pseudowords typically display lower response accuracy compared to words, often in association with an exaggerated presence of fast impulsive errors, which would reflect lexical capture phenomena driven by the lexical activation produced by word-like pseudoword stimuli (Scaltritti et al., 2021; Scaltritti et al., 2023a, 2023b). This pattern resembles the one observed in conflict tasks, where the prepotent task-irrelevant feature triggers response capture phenomena, thereby exaggerating the rate of fast errors. Under these circumstances, the monitoring system(s) may implement stronger monitoring policies on the responses and rely on online control mechanisms to detect and correct potential errors.

Also, compared to words, pseudoword responses reveal a higher rate of partial errors (Scaltritti et al., 2023a, 2023b), akin to incompatible versus compatible conditions within conflict tasks. Partial errors consist of the covert EMG activations of the incorrect response hand that are detected and corrected before completion. The phenomenon maps directly onto the construct of online response control (Burle et al., 2002), by revealing online corrective mechanisms of response execution, which may be more frequently invoked for pseudoword responses, in order to overcome fast impulsive errors driven by lexical capture.

Importantly, online response control processes have been linked to the duration of MTs. For example, prolonged MTs have been highlighted in the case of incorrect responses, reflecting increases in the execution processes determined by response inhibition (Allain et al., 2004). More generally, the duration of peripheral processes of motor-response execution seems directly related to the efficiency of response control and action monitoring, by determining the amount of time available for correcting erroneous response tendencies (Ramdani et al., 2021; Spieser et al., 2017). More error-prone items featuring a higher degree of uncertainty, possibly due to the absence of a corresponding trace in memory’s stores (e.g., pseudowords), may thus call for prolonged MTs to accommodate the implementation of enhanced response-control processes. In this scenario, the lexicality effect detected on measures of response execution would be determined by response control dynamics, with a lengthening of MT for pseudowords compared to words.

To tackle the hypothesis that lexicality effects on MTs originate from response monitoring, the present study implemented a lexical decision task and a manipulation of the speed-accuracy tradeoff (SAT), aimed at influencing the chances for online response control. SAT phenomena (Heitz, 2014) reflect the fact that when instructions emphasize response speed, reaction times (RTs) decrease at the expense of accuracy. Differently, a focus on response accuracy slows down the RTs and increases the proportion of correct responses. SAT-induced variations in RTs are equally reflected in both the PMT and the MT components of RTs (Spieser et al., 2017; Steinemann et al., 2018). Importantly, SAT manipulations can modulate the response control policy in at least two different ways. On the one hand, SAT instructions can influence the efficiency of error monitoring systems (Gehring et al., 1993) – which would be reduced under speed pressure, possibly in relation to the more general hindering of executive cognitive control under speed pressure (Burle et al., 2014; Wylie et al., 2007) – and enhanced when accuracy is emphasized. On the other hand, SAT manipulations – by modulating the duration of the motor-responses themselves – can alter the time available for controlling incorrect response tendencies (Ramdani et al., 2021; Spieser et al., 2017), which would again be reduced under speed stress, and enhanced when responses are biased towards accuracy.

Under the two hypotheses that (a) MTs capture (among other things) decision-informed online response control, and (b) the lexicality effect on MTs reflects an enhanced online control for pseudo-items, the prediction would be that lexicality effects on MTs are reduced, or even eliminated, when speed instructions limit response control processes, either by directly hindering their efficiency (Gehring et al., 1993) or by reducing the time during which these processes may take place and unfold (Ramdani et al., 2021). Instead, the lexicality effect may be enhanced when accuracy is emphasized. A neutral condition, in which speed and accuracy were equally emphasized, was administered to serve as a baseline.

Other than relying on the chronometric measures described above, we sought converging evidence via measures of response accuracy and EMG curves. With regard to accuracy, we assessed the presence of both partial and fast errors. Partial errors reflect covert EMG activations of the incorrect response hand that are detected and corrected before completion, thus directly mapping onto the construct of online response control (Burle et al., 2002). Fast overt errors were assessed by examining conditional accuracy functions (CAFs), representing variations in accuracy as a function of response latencies. In this context, fast errors are more likely to occur under speed instructions (Band et al., 2003; Wylie et al., 2009; van den Wildenberg et al., 2010; for limitations concerning this approach and alternative methods, see Servant et al., 2018), reflecting the limited ability of the decision-system to prevent impulsive responses. Complementarily, we also considered variations of incorrect response activations as a function of response latency (conditional incorrect activations functions, CIAFs; Fluchère et al., 2018; Ramdani et al., 2015). A higher rate of fast incorrect activations (i.e., partial or overt errors) can indeed reveal impulsive response tendencies and increased propension towards errors, irrespective of the ability to correct them online. Finally, we also considered measures of correction likelihood (Burle et al., 2002), by estimating the probability with which incorrect activations are successfully corrected. Taken together, these indexes provide a comprehensive description with respect to the control policy adopted by the decision system under the different experimental conditions and the related ability to prevent or correct errors via mechanisms of online response control.

Measures related to the EMG curve were used to further qualify the propagation of decisional effects on motor responses and to check the consistency of our SAT phenomena with those reported in the field of perceptual decisions (Spieser et al., 2017). We focused both on the amplitude of the EMG curves, reflecting the strength of the motor command and the intervention of online inhibitory mechanisms of response control (Allain et al., 2004), and on the slope of their rising flanks, which captures the synchronization of the motor units’ discharge (Allain et al., 2004; Possamaï et al., 2002) and thus qualitative differences in terms of cortico-spinal commands. SAT instructions have been linked with modulations of both parameters (Spieser et al., 2017), and online response control has been more selectively related to changes in amplitude (e.g., Allain et al., 2004).

## Method

### Participants

The determination of sample size followed recent recommendations in the field (Brysbaert, 2019) and previous investigations (Scaltritti et al., 2023a, 2023b). Forty-eight Italian native speakers participated in the experiment (37 females, *M*_*age*_ = 22.33 years; *SD*_*age*_ = 3.14 years) and received a 20€ compensation. They all had normal or corrected-to-normal vision and reported no motor impairments and no history of neurological issues or learning disabilities. Following the Edinburgh Handedness Inventory (Oldfield, 1971), all the participants could be classified as pure right-handers (*M* = 88.14, *SD* = 15.47), except for one mixed right-hander (5.3). Data from four participants were discarded for technical issues during the experiment (one due to a faulty electrode; one due to an unexpected shutdown of the laptop administering the experimental procedure; two due to amplifier failures). The final sample thus included 44 participants. All the procedures were approved by the ethics committee of the University of Trento (protocol number 2020–028), and participants provided signed informed consent. Raw data and materials are publicly available via the Open Science Framework at https://osf.io/ju9xw/

### Stimuli

Three different sets of 250 words were selected from the PhonItalia database (Goslin et al., 2014), and three sets of 250 pseudowords were created. Within each set, words and pseudowords were comparable in terms of the psycholinguistic variables listed in Table 1. Words and pseudowords were comparable, for the same variables, across the different sets. The word *bronco* (bronchus) was inadvertently included among the pseudowords and thus removed from all the analyses.

**Table 1:** Psycholinguistic variables for the three sets of stimuli used in the experiment

	Set 1			Set 2			Set3		
Variables	Words	PWs	t	Words	PWs	t	Words	PWs	t
Frequency (log)	4.38	-		4.23	-		4.35	-	
Familiarity	6.82	-		6.85	-		6.88	-	
Imageability	7.21	-		7.22	-		7.14	-	
Concreteness	6.52	-		6.59	-		6.36	-	
Valence	5.80	-		5.62	-		5.70	-	
Arousal	5.49	-		5.44	-		5.48	-	
No. of letters	7.01	7.01	0.02	6.92	6.92	0.00	6.86	6.86	0.00
Orthographic N	3.78	4.09	-0.61	3.83	4.66	-1.50	4.02	4.57	-1.04
OLD20	2.03	2.11	-1.47	2.02	2.09	-1.29	2.03	2.11	-1.35
Bigr. freq. sum	709,339	688,062	0.75	708,647	691,673	0.64	676,783	669,684	0.26
Bigr. freq. mean	116,141	111,090	1.59	118,391	114,769	1.21	114,139	111,421	0.86

PWs = pseudowords; Orthographic N = number of orthographic neighbors; OLD20 = orthographic Levenshtein distance to the twenty closest neighbors (Yarkoni et al., 2008); Bigr. freq. sum = summed bigram frequency; Bigr. freq. mean = mean bigram frequency. For words, surface variables were extracted from the PhonItalia database (Goslin et al., 2014). Familiarity, concreteness, imageability, valence, and arousal scores were taken from the Italian adaptation (Montefinese et al., 2014) of the Affective Norms for English Words database (Bradley & Lang, 1999). For pseudowords, the number of orthographic neighbors and OLD20 were computed on the PhonItalia database using the *vwr* package (Keuleers, 2013) in R. Bigram frequency variables were drawn from the same database using a custom-made script. The *t*-values result from independent-sample two-tailed *t*-tests

### Apparatus and procedures

The experiment began after the collection of participants’ demographic and health-related information and installation of EMG electrodes. The experimental procedure was controlled via the E-Prime 2 software (version 2.0.10.356, Psychology Software Tools) running on a laptop. Participants sat in front of the screen at about 50 cm, with two cylindric handheld buttons (connected to a Black Box Toolkit module), and were instructed to classify letter strings as words or pseudowords using their thumbs to perform button presses. The response force threshold for the handheld buttons corresponded to ~ 490–550 g.

The experiment was divided into three main blocks. In the baseline block, response accuracy and speed were equally emphasized. In the other blocks, participants were asked to respond very quickly (speed condition) even at the expense of response accuracy, or very accurately (accuracy condition) even at the expense of response speed. Other than by verbal instruction, the SAT manipulation was implemented using different response deadlines (see below). Halfway through each block, the stimulus (word vs. pseudoword) response (right vs. left hand) mapping was reversed, to ensure within each participant an equal number of responses between the two hands within each category of stimuli. The first block always corresponded to the baseline condition, to avoid potential carryover influences from SAT manipulations. The order of the other two blocks (speed and accuracy conditions) was counterbalanced across participants. Assignment of the three sets of word and pseudoword stimuli, as well as the order in which the two stimulus–response mappings were alternated within each block, was counterbalanced across participants. Before each block and before each inversion of the stimulus–response mappings, 32 practice trials were administered to familiarize participants with the task configuration. Stimuli for practice trials were not part of the experimental set. Participants could take self-terminated breaks every 125 trials. The whole experimental session lasted about 150 min.

Stimuli were presented in 25-pt Courier New font, in black against a gray background (RGB: 200, 200, 200). Trials started with a fixation cross ( +) with a randomly sampled duration (700, 750, 800, 850 ms). Then the stimulus appeared and remained on the screen until participants’ response or for a maximum of 1,500 ms in the baseline condition, 800 ms in the speed condition, and 5,000 ms in the accuracy condition. Error feedback (*ERROR*, in red) was displayed for 500 ms immediately after incorrect responses and, similarly, a *NO RESPONSE* message was displayed for 500 ms when no response was delivered within the deadline. A blank screen lasting 400 ms served as an inter-trial interval.

### EMG recording and processing

EMG activity was acquired via an eego sports system (ANT Neuro) with a sampling rate of 1,000 Hz, using two pairs of bipolar electrodes installed on the thenar eminences of both hands. The ground electrode was placed on the pisiform bone of the right wrist. Before the installation of the electrodes, isopropyl alcohol and a mildly abrasive gel (Nuprep, Weaver and Company, Aurora, CO, USA) were applied for skin preparation. The EMG signal was monitored online, and participants were asked to relax when tonic noise surfaced. Offline processing was performed using EEGLAB (version 14_1_2b; Delorme & Makeig, 2004), ERPLAB (Lopez-Calderon & Luck, 2014), and FieldTrip (version 20,190,203; Oostenveld et al., 2011) functions in MATLAB (version 2018b, MathWorks Inc., Natick, MA, USA), as well as custom-made scripts.

A 10-Hz order 2 Butterworth high-pass filter and a 50-Hz notch filter were first applied. From the continuous filtered signal, we extracted epochs starting 500 ms before stimulus onset and lasting until 1,000 ms after the maximum time allotted for responses (irrespectively of RT) across the different blocks (baseline condition: 2,500 ms; speed condition: 1,800 ms; accuracy condition: 6,000 ms), in order to include the whole EMG burst in the epochs, even for trials featuring the slowest possible responses. Within each epoch, the onset of the EMG signal was determined using the integrated profile method (Liu & Liu, 2016; see also Weindel et al., 2021). The cumulative sum of the absolute values of the EMG trace was first computed and then subtracted from the straight line connecting the first and the last data points (i.e., the cumulative sum of a uniform distribution). The EMG onset corresponded to the sample at which this difference reached the minimum value.

To aid the detection of artifacts and partial responses, in each epoch we identified windows of EMG activity corresponding to the samples in which the signal exceeded the threshold of 3.5 SDs above the mean (absolute) activity in the baseline period (from − 500 to 0 ms). Windows separated by intervals shorter than 25 ms were merged. Windows of activity lasting less than 50 ms and/or beginning after the epoch’s RT were not considered by the algorithm. All the epochs were then visually inspected and we retained only those in which (a) the EMG onset was correctly identified, and (b) the EMG onset was marked in correspondence to the last window of activity before the button-press, thus excluding artifactual data stemming from sudden noise bursts, false-starts, or drifts. On average, 0.02 of the total epochs (*SD* = 0.02) were excluded from all analyses on the basis of these criteria (baseline condition: *M* = 0.02, *SD* = 0.02; speed condition: *M* = 0.01, *SD* = 0.02; accuracy condition: *M* = 0.03, *SD* = 0.03).

The same algorithms were also applied to the channel of the hand not involved in the final button-press to assess partial errors and partial correct responses. Epochs containing one (or more) windows of activity in these channels were highlighted and each epoch was then visually inspected. Partial responses were deemed reliable when the subthreshold activation was visually clear and its onset was accurately marked. Of all the valid trials, partial errors involved 0.05 of the responses (*SD* = 0.04), whereas partial correct responses occurred in 0.01 of the epochs (*SD* = 0.01). As the latter phenomenon was so infrequent, it was not further analyzed.

Epochs time-locked to the EMG onsets (-500 ms to 1,000 ms) of the button-press responses were finally extracted for analyses of the EMG curves.

### Measures

#### Chronometric measures

Only trials with pure correct responses (errors, partial errors, and partial correct responses were excluded) and with accurate onsets were analyzed. On average, the analyses included 1,285 (0.86) trials per participant (*SD* = 105). Other than RTs, we considered measures of PMT (the interval from stimulus onset until the onset of the EMG burst) and MT (time elapsing from EMG onset until button-press).

#### Response accuracy

Accuracy analyses included correct versus incorrect responses, irrespective of the presence of covert EMG activation in the other hand. Although trials with no responses (0.70% of the total trials) are usually considered errors, in the present experiment the large majority of these cases were clustered in the speed block (2.09% of the trials in this condition; neutral = 0.32%; accuracy = 0.04%). As the failure to meet the response deadline may reflect a different underlying generative process compared to the execution of an overt incorrect response, we focused on those trials in which responses were delivered within the allotted time.

Conditional accuracy functions (CAFs), indexing variations in accuracy as a function of RTs, were computed by partitioning trials (within each participant and condition) into five quantiles as a function of RTs. Quantiles were treated as a fixed effect in the analyses. Conditional incorrect activations functions (CIAFs) were computed in a similar way, except that the latency of partial errors was included in the data, in order to measure the variations in the proportion of incorrect EMG activations as a function of (partial) response latency.

Analyses of partial errors focused on correct responses to assess variations in the likelihood of covert incorrect responses as a function of experimental conditions. As for the accuracy analyses, trials with no responses were discarded.

Finally, correction likelihood was assessed by focusing on trials featuring overt or covert error responses (i.e., full and partial errors). Accuracy was then assessed as a function of experimental conditions to estimate the likelihood that an incorrect EMG activation would be successfully corrected online. The analysis was implemented to better characterize response control processes over and above variations in terms of accuracy and/or partial responses across SAT conditions.

#### EMG curve

Epochs time-locked to the onset of the EMG bursts were separately averaged for each participant and each condition. From the resulting EMG curves, two measures were extracted: mean amplitude and the slope of the rising flank. For each curve, the mean amplitude was computed in the interval between time 0 until the sample in which the decreasing flank of the curve returned to a value corresponding to 30% of its absolute peak. The rationale for this procedure was to include a constant (and large) part of the EMG curves irrespective of the huge variability in their shape across participants and conditions. Following Spieser et al. (2017), the slope of the rising flank was estimated, for each EMG curve, by fitting a linear regression to the samples included between 0 and 30 ms and extracting the resulting coefficient.

#### Statistical analyses

Chronometric measures were analyzed using linear mixed-effects (LME) models. Binomial measures of response accuracy and partial errors were analyzed via generalized mixed-effects models. Indexes of the EMG curves were analyzed using ANOVAs.

For (generalized) LME models, fixed effects (SAT: neutral, accuracy, speed; Lexicality: words vs. pseudoword) were considered significant when their corresponding *t* or *z* value was larger than |2|. The random effect structure of maximal complexity (Barr et al., 2013) was fitted, including random slopes for all the fixed terms and their correlations with the intercepts. The structure was progressively simplified when models failed to converge (e.g., Bates et al., 2018; Matuschek et al., 2017), first by fitting zero-correlation models, then by removing random slopes (or intercepts) associated with the smallest amount of variance.

For both the CAF and CIAF analyses we assessed the interaction between quantiles, SAT condition, and lexicality together with potential nonlinear relationships using second-order orthogonal polynomials to fit the quantile variables. Nonlinear terms were retained only when they increased goodness of fit, as estimated via a loglikelihood test. To aid model convergence, for these analyses the random-effect structure was limited to random intercepts. Also, for all the analyses featuring a dichotomous dependent variable, the maximum number of allowed iterations was increased (2e^5^) and a different optimization algorithm for the second stage optimization of the models was used (*bobyqa* instead of *Nelder-Mead*). For ANOVAs, Greenhouse–Geisser correction (Greenhouse & Geisser, 1959) to the degrees of freedom was applied when sphericity assumptions were violated.

For all the analyses, potential follow-up pairwise comparisons were conducted on estimated marginal means. A Bonferroni correction was applied to the resulting *p*-values when multiple comparisons were computed on the same set of data, to control for Type I error inflation. The analyses were conducted using the lme4 (version 1.1.27.1; Bates et al., 2015) and the afex packages (version 28.1; Singman et al., 2021) in R (version 4.2.1; R Core Team, 2015). Figures were made using the ggplot2 package (version 3.3.6; Wickham, 2016) in R or with MATLAB basic functions.^1^

## Results

### Chronometric measures

For all models conducted on chronometric measures, detailed parameters of the fixed effects are presented in Table 2 (random effects are reported in the Online Supplementary Materials (OSM) 1, Table S1). In terms of RTs, compared to the neutral condition, responses were faster under speed instructions and slower under accuracy instructions. There was a clear lexicality effect, with slower RTs for pseudowords. The lexicality effect was reduced in the blocks emphasizing speed and enhanced in those focusing on accuracy. RTs, however, were shorter for words compared to pseudowords in all SAT conditions (neutral: *Est.* = -67.6, *SE* = 3.81, *z* = -17.75, *p* < 0.001; speed: *Est.* = -41.7, *SE* = 3.94, *z* = -10.59, *p* < 0.001; accuracy: *Est.* = -98.2, *SE* = 3.77, *z* = -26.06, *p* < 0.001).

**Table 2:** Parameters of the fixed effects for linear mixed-effects (LME) models of chronometric measures

	RT			PMT			MT		
Fixed effects	Est	SE	t	Est	SE	t	Est	SE	t
Intercept	656.94	11.51	57.07	524.27	9.74	53.82	132.59	5.55	23.89
Lexicality (pseudo.)	67.59	3.81	17.75	61.64	3.68	16.75	5.94	1.24	4.78
SAT (acc.)	195.47	33.68	5.80	172.41	32.21	5.35	23.07	4.53	5.09
SAT (speed)	-119.25	8.64	-13.81	-94.87	7.03	-13.50	-24.37	3.46	-7.04
Lexicality (pseudo.) x SAT (acc.)	30.66	3.70	8.28	29.71	3.60	8.26	1.01	2.25	0.45
Lexicality (pseudo.) x SAT (speed)	-25.91	3.87	-6.70	-25.058	3.75	-6.67	-0.81	1.48	-0.55

RT = reaction time; PMT = premotor time; MT = motor time; SE = standard error; pseudo. = pseudoword; acc. = accuracy; SAT = speed-accuracy tradeoff

For PMTs, the results mimicked those on RTs. Compared to the neutral condition, lexicality effects were again smaller under speed instructions, and larger under accuracy instructions. Lexicality effects were still significant across all SAT conditions (neutral: *Est.* = -61.6, *SE* = 3.68, *z* = -16.75, *p* < 0.001; speed: *Est.* = -36.6, *SE* = 3.8, *z* = -9.62, *p* < 0.001; accuracy: *Est.* = -91.3, *SE* = 3.64, *z* = -25.08, *p* < 0.001).

**Fig. 1:**
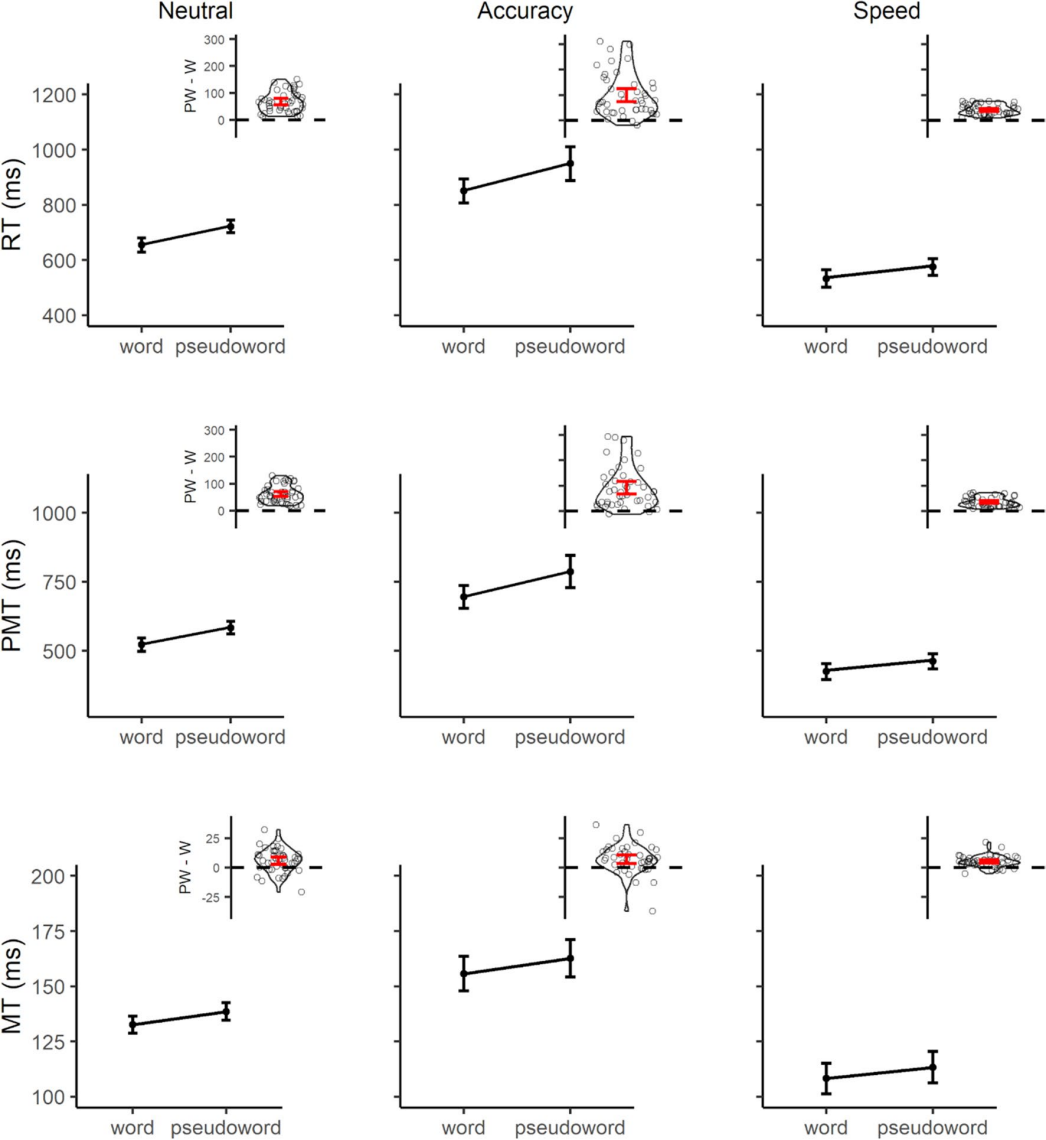
Results for reaction time (RT; first row), premotor time (PMT; second row), and motor time (MT, third row), as a function of SAT instructions (Neutral, first column; Accuracy, second column; Speed, third column). Points represent empirical means, with error bars highlighting 95% confidence intervals, adjusted for within-participants variables following Morey (2008). Lines represent models’ estimates. Inset plots provide an overview of the lexicality effect across participants (PW = pseudoword; W = word). Points represent individual difference-scores between pseudowords and words in the corresponding measure, with the violin-plot describing their distribution. Red error-bars highlight 95% confidence-interval of the mean effect for the whole sample

For MTs, instead, the lexicality effect does not seem to be modulated as a function of SAT regime. Given the theoretical importance of this null effect, we sought confirmation via Bayes factor (BF) estimation. The Bayesian Information Criterion (BIC) of the model featuring the interaction was subtracted from the one featuring additive terms to compute the delta BIC. The BF was then approximated following the formula exp(deltaBIC/2) (Raftery, 1995), and corresponded to BF = 0.00048. The lexicality effect was indeed significant and remarkably similar irrespectively of SAT conditions (neutral: *Est.* = -5.94, *SE* = 1.24, *z* = -4.78, *p* < 0.001; speed: *Est.* = -5.13, *SE* = 1.76, *z* = -2.91, *p* = 0.011; accuracy: *Est.* = -6.95, *SE* = 2.45, *z* = -2.84, *p* = 0.013). Results are summarized in Fig. 1.

#### Response accuracy

For the analysis of accuracy, detailed parameters of the fixed effects are presented in Table 3 (for random effects, see OSM 1, Table S2). Compared to the neutral blocks, accuracy was significantly lower under speed instruction and higher in the accuracy condition. Unexpectedly, we observed a reversed effect of lexicality, with higher accuracy for pseudowords, although numerically the difference seems negligible (words = 0.939; pseudowords = 0.944). Moreover, the direction of the difference between words and pseudowords assumes the standard direction in the speed blocks (see also OSM 2, Fig. S1). Follow-up comparisons, however, revealed that the lexicality effect failed to reach conventional significance in the different SAT regimes (neutral condition, *Est*. = -0.27, *SE* = 0.12, *z* = -2.34, *p* = 0.06; accuracy, *Est*. = -0.23, *SE* = 0.13, *z* = -1.72, *p* = 0.26; speed, *Est*. = 0.10, *SE* = 0.10, *z* = 0.99, *p* = 0.96).

**Table 3:** Parameters of the fixed effects for generalized linear mixed-effects (GLME) models on response accuracy, partial errors, and correction likelihood

	ACC			PE			CL		
Fixed effects	Est	SE	z	Est	SE	z	Est	SE	z
Intercept	3.95	0.13	29.73	-3.17	0.13	-23.93	0.54	0.15	3.52
Lexicality (pseudo.)	0.27	0.12	2.34	-0.04	0.07	-0.55	0.23	0.13	1.80
SAT (acc.)	0.84	0.13	6.59	-0.75	0.13	-5.79	0.13	0.18	0.75
SAT (speed)	-1.14	0.11	-10.81	0.29	0.10	2.80	-0.97	0.15	-6.59
Lexicality (pseudo.) x SAT (acc.)	-0.04	0.12	-0.38	0.09	0.10	0.94	0.23	0.20	1.18
Lexicality (pseudo.) x SAT (speed)	-0.37	0.09	-4.13	0.08	0.08	1.00	-0.36	0.15	-2.41

ACC = response accuracy; PE = partial errors; CL = correction likelihood; SE = standard error; pseudo. = pseudoword; SAT = speed-accuracy tradeoff; acc. = accuracy. Values are on the log-odds scale

**Fig. 2:**
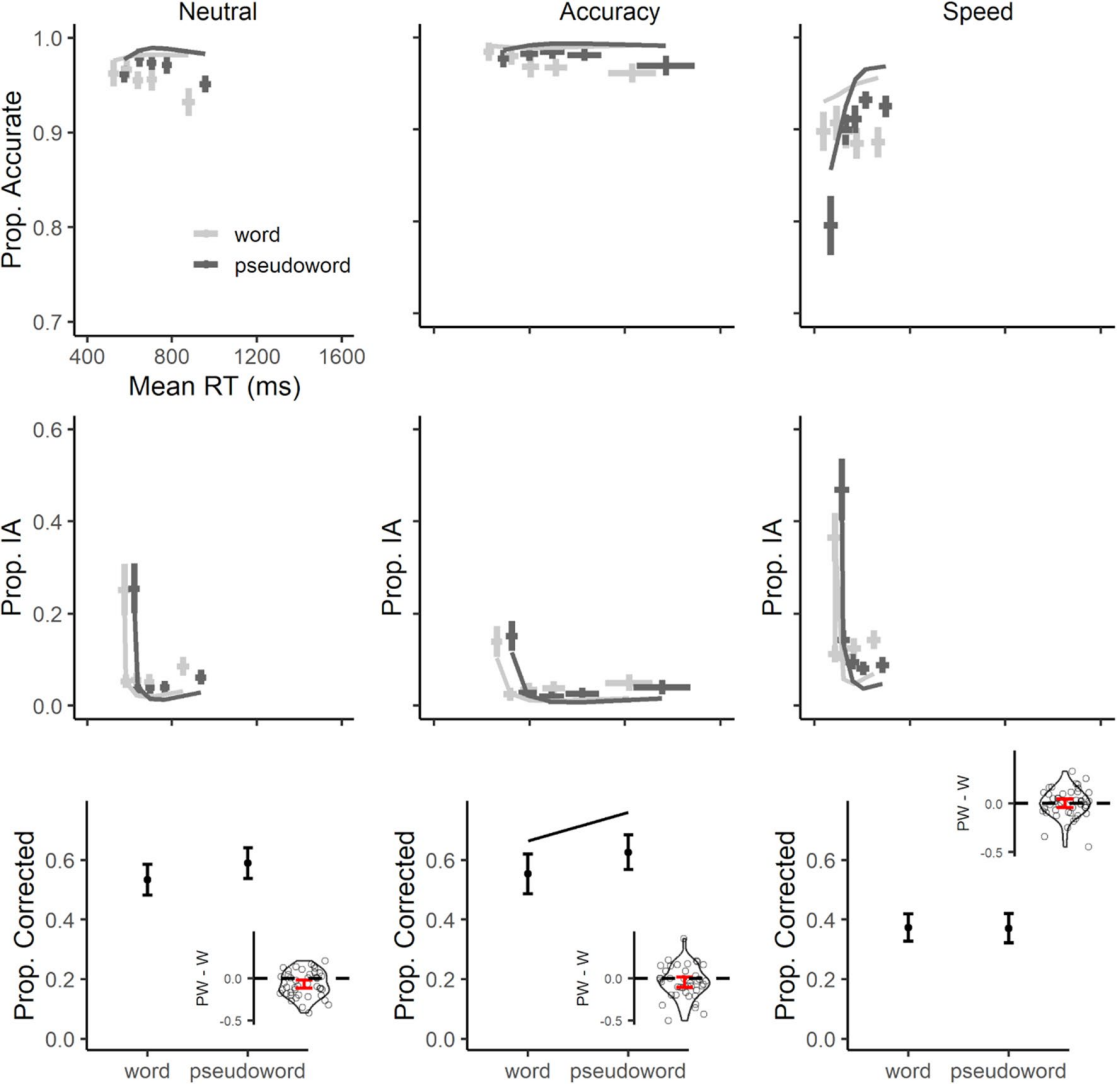
Results for CAFs (first row), CIAFs (second row), and correction likelihood (third row) as a function of SAT instructions (Neutral, first column; Accuracy, second column; Speed, third column). For CAFs and CIAFs, points represent empirical means, with vertical error bars highlighting 95% confidence intervals, adjusted for within-participants variables following Morey (2008). Horizontal error bars reflect 95% confidence interval of the average reaction time (RT) within each quantile. Lines represent models’ predicted means. Prop. IA = Proportion of Incorrect Activations. Within correction likelihood plots, points represent empirical means, with error bars highlighting 95% confidence intervals, adjusted for within-participants variables following Morey (2008). Lines represent models’ estimates (no line appears when the lexicality effect was not significant). Inset plots provide an overview of the lexicality effect across participants (PW = pseudoword; W = word). Points represent individual difference-scores between pseudowords and words in the corresponding measure, with the violin-plot describing their distribution. Red error-bars highlight 95% confidence-interval of the mean effect for the whole sample

For CAF analyses, the inclusion of second-order orthogonal polynomials for the quantile variable increased goodness-of-fit, χ^2^ (2) = 59.63, *p* < 0.001, and improved the modeling of the interaction between quantiles and other factors (OSM, Tables S2 and S3). In baseline and accuracy blocks, most errors were associated with the slowest latencies, indicating failures in stimulus identification. However, the primary finding from these analyses was a significant increase in fast, impulsive errors under speed instructions, especially for pseudowords (Fig. 2). The enhancement of fast errors for pseudowords was not clearly detectable under the other SAT conditions, in contrast with previous observations (Scaltritti et al., 2021; Scaltritti et al., 2023a, 2023b).

Even for CIAF analyses, fitting a second-order orthogonal polynomial for the quantile variable increased goodness-of-fit, χ^2^ (2) = 13.52, *p* = 0.001, and contributed to a better modeling of the interactions between quantiles and other variables (OSM 1, Tables S2 and S4). Interestingly, fast activations of incorrect responses were present across all SAT regimes, although clearly exaggerated under speed pressure. Additionally, under speed instructions, the enhanced rate of impulsive incorrect EMG activations is magnified for pseudowords compared to words (Fig. 2).

Compared to the baseline condition, partial errors were more likely to occur under speed instructions and reduced when accuracy was emphasized (Table 3). Contrary to previous observations (Scaltritti et al., 2023a, 2023b), there were no clear differences as a function of stimulus lexicality. Additionally, there was no evidence of any interaction between lexicality and SAT (*z*s < 1.1, see Table 3; random effects in OSM 1, Table S2).

Finally, the analyses of correction likelihood revealed that online corrections were reduced under speed instructions, compared to the baseline condition. Crucially, there was a significant decrease in the likelihood of correcting incorrect activations for pseudoword responses in the speed blocks (Table 3; random effects in OSM 1, Table S2).

#### EMG curve

**Fig. 3:**
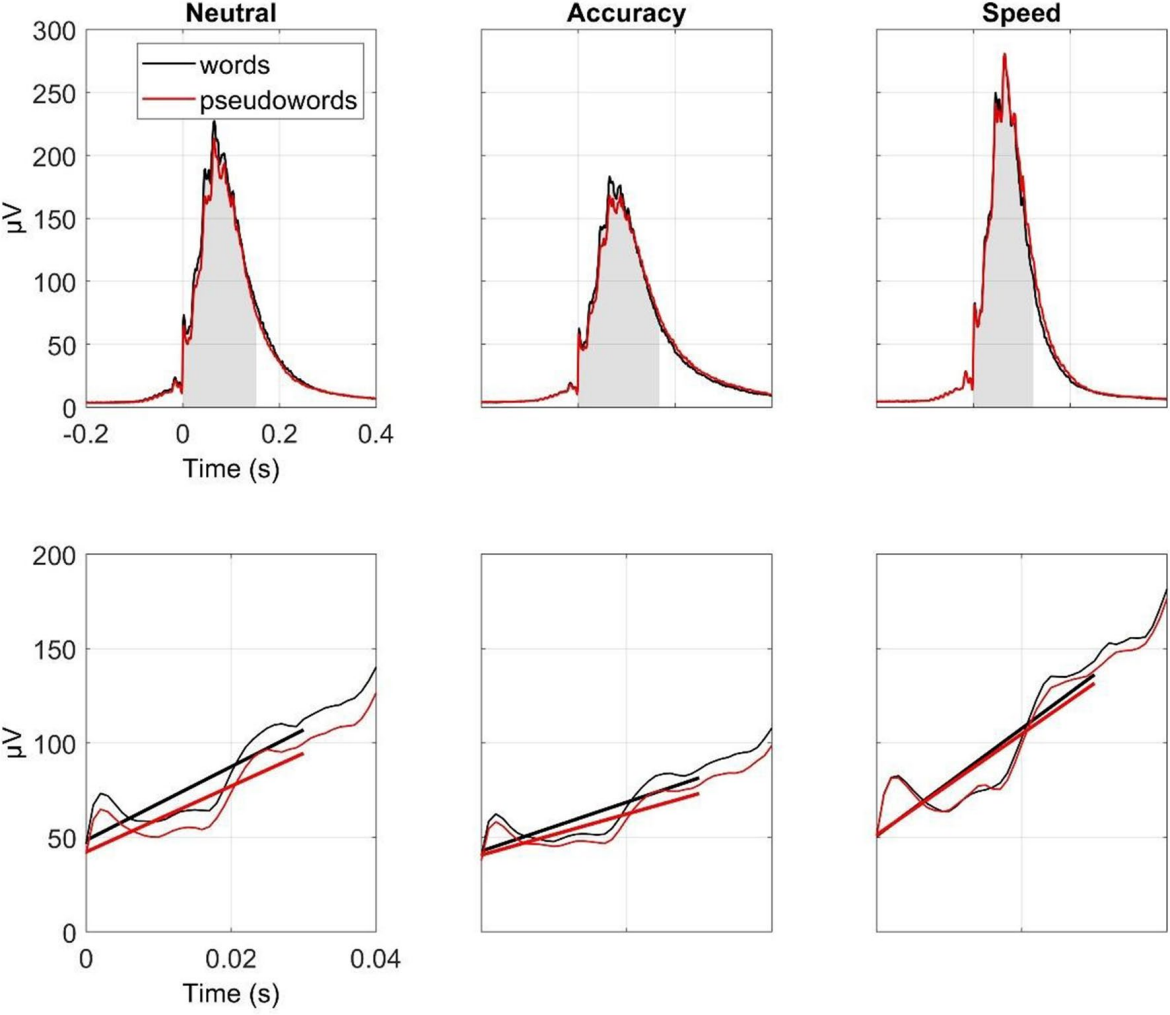
Results for electromyographic (EMG) curve amplitude (first row), and slope (second row) as a function of speed-accuracy tradeoff (SAT) instructions (Neutral, first column; Accuracy, second column; Speed, third column). In the first row, the shaded area corresponds to the median interval tested in the different conditions (see text for details). In the second row, the thick lines represent the estimated slope of the EMG curves in the time-interval between 0 and 30 ms

The analyses on the EMG curves (Fig. 3) were performed on epochs featuring pure correct responses (errors, partial errors, and partial correct responses were excluded) and accurate onsets. On average, the analyses included 1,287 (0.86) epochs per participant (*SD* = 105; 0.07). Concerning the amplitude of the EMG curves, the main effects of SAT, *F* (1.84, 79.01) = 31.50, *MSE* = 2211.06, *p* < 0.001, *η*^*2*^ = 0.08, and lexicality, *F* (1, 43) = 4.93, *MSE* = 320.22, *p* = 0.032, *η*^*2*^ = 0.001, were significant. The interaction failed to reach conventional significance, *F* (1.36, 58.63) = 2.62, *MSE* = 374.22, *p* = 0.100, *η*^*2*^ =  < 0.001. Compared to the baseline condition, EMG curves of the accuracy condition were significantly smaller, *Est.* = 20.0, *SE* = 6.72, *t* (43) = 2.97, *p* = 0.01, whereas those obtained under speed instructions were larger, *Est.* = -33.4, *SE* = 5.90, *t* (43) = -5.66, *p* < 0.001. Concerning the lexicality effect, the amplitude of the EMG curve was larger for words compared to pseudowords, *Est.* = 4.89, *SE* = 2.20, *t* (43) = 2.22, *p* = 0.032.

Concerning the slope of the rising flank, again the main effects of SAT, *F* (1.60, 68.95) = 72.83, *MSE* = 0.94, *p* < 0.001, *η*^*2*^ = 0.22, and lexicality, *F* (1, 43) = 19.20, *MSE* = 0.13, *p* < 0.001, *η*^*2*^ = 0.007, were significant, with no interaction, *F* (1.83, 78.57) = 0.12, *MSE* = 0.11, *p* = 0.874, *η*^*2*^ < 0.001. The slope of the neutral condition was steeper than under accuracy instructions, *Est.* = 0.66, *SE* = 0.10, *t* (43) = 6.50, *p* < 0.001, and less steep compared to the speed condition, *Est.* = -0.91, *SE* = 0.13, *t* (43) = -7.17, *p* < 0.001. The slopes were significantly steeper for word compared to pseudoword responses, *Est.* = 0.19, *SE* = 0.04, *t* (43) = 4.38, *p* < 0.001.

Concerning these analyses, two issues warrant some caution in their interpretation. First, shorter MTs would reduce the jittering of the physiological responses, thereby inflating the steepness and the amplitude of the average EMG curve over and above any underlying difference in terms of strength and efficiency of the motor command (but see, e.g., Allain et al., 2004, for evidence that shorter MTs are not necessarily associated with a steeper rising flank of the average EMG burst). Second, EMG curves revealed values above 0 at the time of the (estimated) onset, suggesting a slight overestimation of the onset times, which seems to be absent when EMG onsets are scored/corrected manually (e.g., Allain et al., 2004; Spieser et al., 2017).

## Discussion

The experiment aimed at specifying the functional characterization of the decisional component reflected in motor-response execution. We assessed its potential links with online response-control mechanisms, which operate during the unfolding of motor responses to both overcome incorrect response tendencies and detect and correct errors (e.g., Burle et al., 2002, 2014; Fluchère et al., 2018; Ramdani et al., 2013; Spieser et al., 2015; Wylie et al., 2007, 2010). In the context of a lexical decision task, we assessed potential variations in the decisional modulation of the motor stage (i.e., the lexicality effect) across different decisional and response-control policies induced by SAT manipulations. Previous evidence suggests that speed pressure may alter response control, either by directly hindering the efficiency of the monitoring system (e.g., Gehring et al., 1993; Taylor et al., 2007) or – indirectly – by reducing the duration of motor-response execution (e.g., by modulating the excitability of motoneurons). This, in turn, reduces the chances of fully implementing online control over the ongoing response (Ramdani et al., 2021; Spieser et al., 2017). Although our experiment is not able to distinguish between these two possibilities, they both predict SAT-induced modulations of the lexicality effect on MTs, if the latter represents a byproduct of online control processes. However, in the present experiment, the lexicality effect at the motor level was impervious to any SAT-induced modulations.

The SAT manipulation revealed the standard pattern of findings: Compared to the neutral condition, both RTs and accuracy decreased when speed was emphasized and increased when the instructions focused on accuracy. Results from MTs mirrored those on RTs (and PMTs). The analysis of the EMG curves also replicated previous findings (Spieser et al., 2017), as both the amplitude and the slope of the rising flanks of the EMG curves were enhanced when speed was emphasized and reduced when instructions focused on accuracy.

Importantly, results from accuracy measures supported the notion that SAT affected online response control. The analysis of conditional incorrect activation functions (CIAFs; Fluchère et al., 2018; Ramdani et al., 2021) revealed that, although fast incorrect activations could be identified across all SAT regimes, their presence was magnified under speed pressure. This speed-induced increase of fast impulsive (incorrect) responses was further exaggerated for pseudowords compared to words, which would become more prone to lexical capture phenomena. Of note, as indicated by conditional accuracy functions, the enhanced rate of impulsive errors for pseudowords induced by speed instructions could be counteracted only in part by response control mechanisms. Whereas fast incorrect activations were almost eliminated under baseline and accuracy conditions (i.e., CAFs revealed no clear trace of fast errors), the difference between words and pseudowords in terms of fast errors remained distinctly discernible within speed blocks, suggesting that only a fraction of the impulsive incorrect activations could be controlled. Consistently, correction likelihood for pseudowords significantly decreased under speed pressure compared to the baseline condition. Therefore, speed instructions seem to both enhance the likelihood of impulsive responses – particularly for pseudoword stimuli – and reduce the correction likelihood of (fast) incorrect response activations, possibly because the reduction in motor time would leave less room for an online response control process (e.g., Ramdani et al., 2021; Spieser et al., 2017) or because speed stress impairs the monitoring system itself (e.g., Gehering et al., 1993).

In this scenario, we deem it surprising that the lexicality effects on MTs remained stable across SAT conditions. None of the phenomena that can be related to variations in response control seemed to necessarily co-occur with the displaying of lexicality effects on motor responses. Pseudowords consistently revealed longer MTs compared to words irrespective of their predisposition towards incorrect response activations (in the form of overt, fast, and/or partial errors) across the different SAT regimes. Additionally, even the SAT-induced modulations of the overall MTs failed to affect the lexicality effect on motor-response duration, as it remained virtually unchanged both when MTs were lengthened by accuracy-focused instructions and when reduced under speed stress. In other words, although variations in MTs should inevitably alter the time allowed for online control processes to operate (e.g., Ramdani et al., 2021), the lexicality effect remained constant, thus pointing towards independence from action monitoring dynamics.

The lexicality effects detected on indexes of motor-response execution suggest that, irrespective of varying decision and control policies, the deliberation concerning the lexical status of the stimulus has not yet reached a definitive commitment prior to response initiation. This is also supported by the analysis of the EMG curves, showing that both the amplitude and the slope of the curves’ rising flank are enhanced for words compared to pseudowords. This pattern may point to qualitative differences in the motor command, and in particular to a different synchronization in terms of motor units discharge (Allain et al., 2004; Hasbroucq et al., 1995; Possamaï et al., 2002), rather than to an online control mechanism which would more selectively affect the amplitude parameter, signaling the intervention of inhibitory mechanisms after the motor command has been issued (Allain et al., 2004; Rochet et al., 2014; Smigasiewicz et al., 2020).

The interpretations of the different measures presented so far clearly borrow from the literature on conflict effects. Although the parallel between the dynamics triggering the recruitment of response control processes in lexical decision and conflict tasks can be just partial, we believe it is tenable. Conflict tasks are inherently characterized by a prepotent task-irrelevant dimension (e.g., the stimulus location in the Simon paradigm) directly prompting a response conflict. In lexical decision, a similar factor can be identified in the partial lexical activation produced by pseudowords, which would drive the system towards an incorrect word response. Although the latter may not represent a prepotent response tendency, it triggers lexical capture phenomena (e.g., Coltheart et al., 2001), which are associated with both the exaggerated presence of fast errors and the enhanced rates of partial errors for pseudoword stimuli (Scaltritti et al., 2021; Scaltritti et al., 2023a, 2023b). Lexical capture in lexical decisions may indeed parallel response capture phenomena induced by prepotent yet task-irrelevant stimulus features within conflict tasks.

Concerning the more general issue of the functional characterization of the motor components of decision making, lexical decision tasks may offer a complementary view to the perspectives offered by perceptual and conflict tasks. On the one hand, lexical decision is critically grounded in conceptual and symbolic information drawn from memory, rather than on perceptual evidence. On the other hand, as previously argued (e.g., Dufau et al., 2012; Grainger & Jacobs, 1996), the information driving pseudoword responses may require a specific conceptualization. Binary decision tasks usually require mapping different stimuli or features (e.g., red vs. green shapes; left- vs. right-pointing arrows; left- vs. right-moving dots) onto two alternative responses. For pseudoword responses, the decisional dynamics may be harder to grasp, as the decision concerns items for which there is no previous representation in long-term memory and, hence, no stored identity. This may introduce some notable differences in terms of decision-making, and the functional characterization of its motor components. It is, however, important to note that, even in the context of a perceptual decision-making task, SAT phenomena failed to reveal any interaction with a manipulation of visual contrast, despite both revealing sizeable simple effects at the level of MTs (Weindel et al., 2021). These data point to a convergence between perceptual decision making and lexical decision on the notion of independence between SAT and other decision-related manipulations.

With respect to the functional characterization of motor decisional components, if we exclude online response control from the potential determinants of the lexicality effect on MTs, we should endorse the possibility that lexical decisions are not terminated before response initiation. The “premature” issuing of the responses may be related to an accumulating time-dependent urgency to respond (Cisek et al., 2009), signaling an increasing likelihood of a response as a function of the passing of time that may possibly drive response onset before the lexical decision has been completed. However, SAT manipulations induced with a response deadline (as was the case for the present experiment) enhance urgency (Katsimpokis et al., 2020). Had the lexicality effect been a mere byproduct of an urgency signal, we would have thus expected stronger lexicality effects in the speed blocks, which was clearly not the case.

On the other hand, the lexicality effect across SAT conditions may capture residual differences in terms of decision confidence upon motor-response onset. One possibility is that a dynamically evolving confidence variable shapes motor-response execution in its unfolding. In this scenario, pseudoword responses may feature a decreased level of confidence, reflecting the reduced probability that the unfolding choice is correct, due to the ambiguity of these stimuli featuring no pre-existing representation in long-term memory stores. Although previous work shows that confidence is inversely related to decision RTs, and thus decreases under speed instructions (Moran et al., 2015), recent evidence points toward a limited influence of choice-related SAT on confidence ratings (Herregods et al., 2023).

The notion that decision confidence may be shaped by a dynamic confidence variable reflecting a (continuing) process of evidence accumulation may seem to fall prey to the same empirical issues (i.e., the dissociations between different decisional variables in their ability to affect MTs) that we highlighted for the notion of a single decisional variable accumulating before and after response onset. Note though that decision confidence could be envisaged as a second-order metacognitive process relying on different informational sources compared to first-order decision processes (for discussion, see, e.g., Bang et al., 2019; Desender et al., 2021a, 2021b; Fleming et al., 2018). In other words, decision- and confidence-related evidence accumulation processes may rely on different information, and factors influencing the two may be partially dissociable (e.g., Bang et al., 2019; Fleming et al., 2018). In the present context, for example, information shaping confidence at the time of response might be related to late-occurring verification processes that would perform an additional check on more ambiguous items with no existing representations in memory, such as pseudowords (e.g., Perea et al., 2005). It remains to be evaluated whether these processing dynamics are specific for responses to pseudo-items or can be ascribed to more general exit-strategies for memory searches in which exhaustive search is not a viable option.

Importantly, proposals from models of metacognition suggest that (post-response) confidence is not uniquely determined by the strength of the decision variable, but also by features of the motor output (e.g., Fleming & Daw, 2017). Consistently, Gajdos and colleagues (2019) have demonstrated that partial motor activations surfacing before the onset of the final response are associated with increased subjective confidence within a perceptual decision task, over and above any influence on response accuracy. The result is consistent with the notion that motor activity contributes to metacognitive judgment about decision confidence, which would encompass the whole perception–action cycle (Gajdos et al., 2019; see also Siedlecka et al., 2016, for related evidence beyond perceptual decision making). In the context of the present experiment, we speculate that even chronometric measures of motor-response execution, particularly motor-response duration, may be related to subjective post-response decision confidence. Although the direction of this relationship remains to be empirically tested, we can hypothesize that the increase in MTs for pseudowords may be associated with reduced confidence at the time of response onset, which in turn may be reflected in post-response confidence ratings.

In conclusion, the functional characterization of motor decisional components remains to be fully specified. Measures of response execution such as MTs, in fact, seem to reflect multiple variables ranging from purely biomechanical ones (e.g., threshold force required for the response) to diverse cognitive factors. Concerning the latter, SAT phenomena (Spieser et al., 2017) and, relatedly, online action monitoring (e.g., Ramdani et al., 2021) are amongst the cognitive components that we can trace even at the level of motor-response execution. The lexicality effect in lexical decision, given its independence from the other factors, may map onto further and yet-to-be-specified decisional motor components. Therefore, similar to RTs, variations in chronometric durations for response execution may be linked with different underlying processes, which can hardly be reconciled with the notion of a single decisional variable percolating to the motor stage. In general, the current data suggest that multiple cognitive factors may independently affect motor-response execution, thus hinting at multiple cognitive determinants in the transition to action and motor responses.

## Authors' contributions (CRediT)

Michele Scaltritti: Conceptualization, Methodology, Software, Formal Analysis, Investigation, Data Curation, Writing – Original Draft, Writing – Reviewing and Editing, Visualization; Supervision; Project Administration; Elena Greatti: Investigation; Data Curation, Writing- Reviewing and Editing; Simone Sulpizio: Conceptualization, Methodology, Writing – Original Draft, Writing – Reviewing and Editing, Visualization, Supervision, Project Administration.

## Supplementary Information

Below is the link to the electronic supplementary material.

Supplementary file1 (DOCX 377 KB)

## Data Availability

Scripts for data processing and analysis are available upon request.

## Footnotes

1 Analyses of chronometric measures, CAFs, and correction likelihood were conducted after trimming trials identified as fast guesses (Weindel et al., 2021) on the basis of the exponentially weighted moving average (EWMA) filter developed by Vandekerckhove and Tuerlinckx (2007). The filter was applied via an implementation in R of the Python algorithm developed by Michael Nunez (https://github.com/mdnunez/bayesutils/blob/master/wienerutils.py), using the default parameters provided therein. The main results were fully replicated. The results of chronometric analyses were further replicated on log-transformed measures and Bayesian estimation of linear mixed-effect models using the brms R package (Bürkner, 2017).

## Declarations

### Open Practices Statement

The experiment was not preregistered. Raw data and materials are publicly available via the Open Science Framework at https://osf.io/ju9xw/.
