# Supplementary material for: Decisional components of motor responses are not related to online response control: Evidence from lexical decision and speed-accuracy tradeoff manipulations
Source: Mem Cognit. 2024 Aug 19;53(3):911–25. doi: 10.3758/s13421-024-01619-3 (PMC12052872; doi:10.3758/s13421-024-01619-3)
Supplement: Supplementary file 1 — Supplementary file1 (DOCX 377 KB) [file 13421_2024_1619_MOESM1_ESM.docx]

**Supplementary Materials**

**Supplementary Materials 1**

***Random Effects for (G)LMEs***

For each model of the article, we present the random effect parameters. The pseudo-code provides a specification of fixed- and random-effects structures. The code *1 + effect | participant* points to a random effect modeled as a random slope and its correlation with the random intercept, whereas the code *effect || participant* identifies a random slope without correlation.

**Table S1. Random effects for LMEs of chronometric measures**

| Group | Random Effect |  | Variance | SD | Correlation |
| --- | --- | --- | --- | --- | --- |
| RT ~ SAT * Lexicality + (1 + SAT \| \| participant) + (1 \| item) | | | | | |
| Item | Intercept |  | 2753 | 52.47 |  |
| Participant | Intercept |  | 5513 | 74.25 |  |
|  | SAT: speed |  | 2951 | 54.33 | - |
|  | SAT: accuracy |  | 49607 | 222.73 | - |
| Residual |  |  | 33618 | 183.35 |  |
| PMT ~ SAT * Lexicality + (1 + SAT \| \| participant) + (1 \| item) | | | | | |
| Item | Intercept |  | 2544 | 50.43 |  |
| Participant | Intercept |  | 3878 | 62.27 |  |
|  | SAT: speed |  | 1861 | 43.14 | - |
|  | SAT: accuracy |  | 45360 | 212.98 | - |
| Residual |  |  | 31709 | 178.07 |  |
| MT ~ SAT * Lexicality + (1 + SAT * Lexicality \| \| participant) + (1 \| item) | | | | | |
| Participant | Intercept |  | 1347.98 | 36.71 |  |
|  | SAT: speed |  | 512.87 | 22.65 | - |
|  | SAT: accuracy |  | 889.26 | 29.82 | - |
|  | Lexicality |  | 53.90 | 7.34 | - |
|  | SAT (acc.) X Lex. (pseudo.) |  | 196.27 | 14.01 | - |
|  | SAT (speed) X Lex. (pseudo.) |  | 66.16 | 8.13 | - |
| Item | Intercept |  | 1.60 | 1.26 |  |
| Residual |  |  | 1521.78 | 39.01 | - |

*Note.* RT = reaction time; PMT = premotor time; MT = motor time; SD = standard deviation.

**Table S2. Random effects for GLMEs of accuracy, CAFs, CIAFs, partial errors, and correction likelihood**

| Group | Random Effect |  | Variance | SD | Correlation | |
| --- | --- | --- | --- | --- | --- | --- |
| Accuracy  ACC ~ SAT * lexicality + (1 + SAT + Lexicality \| \| participant) + (1 \| item) | | | | | | |
| Item | Intercept |  | 1.38 | 1.17 |  | |
| Participant | Intercept |  | 0.54 | 0.74 |  | |
|  | Lexicality |  | 0.14 | 0.38 |  | |
|  | SAT: speed |  | 0.32 | 0.56 |  | |
|  | SAT: accuracy |  | 0.37 | 0.60 |  | |
| CAFs  ACC ~ SAT * Lexicality * Quantile, second order polynomial + (1 \| participant) + (1 \| item) | | | | | | |
| Item | Intercept |  | 1.53 | 1.24 |  | |
| Participant | Intercept |  | 0.42 | 0.65 |  | |
| CIAFs  ACC ~ SAT * Lexicality * Quantile, second order polynomial + (1 \| participant) + (1 \| item) | | | | | | |
| Item | Intercept |  | 1.16 | 1.08 |  | |
| Participant | Intercept |  | 0.55 | 0.74 |  | |
| Partial Error ~ SAT * lexicality + (1 + SAT + Lexicality \| \| participant) + (1 \| item) | | | | | | |
| Item | Intercept |  | 0.19 | 0.44 |  | |
| Participant | Intercept |  | 0.65 | 0.81 |  | |
|  | Lexicality |  | 0.03 | 0.18 |  | |
|  | SAT: accuracy |  | 0.46 | 0.68 |  |  |
|  | SAT: speed |  | 0.29 | 0.54 |  |  |
| Correction Likelihood  IA ~ SAT * lexicality + (1 + SAT * Lexicality \| \| participant) + (1 \| item) | | | | | | |
| Item | Intercept |  | 0.83 | 0.91 |  |  |
| Participant | Intercept |  | 0.67 | 0.82 |  |  |
|  | Lexicality |  | 0.04 | 0.20 |  |  |
|  | SAT: accuracy |  | 0.58 | 0.76 |  |  |
|  | SAT: speed |  | 0.50 | 0.70 |  |  |
|  | SAT (acc.) X Lex. (pseudo.) |  | 0.08 | 0.28 |  |  |
|  | SAT (speed) X Lex. (pseudo.) |  | 0.06 | 0.25 |  |  |

*Note.* ACC = accuracy; PE = partial error; IA = incorrect activation; SD = standard deviation.

**Supplementary Materials 2**

***Additional Information for Analyses on Response Accuracy***

**Figure S1**

*Results for the accuracy measures.*


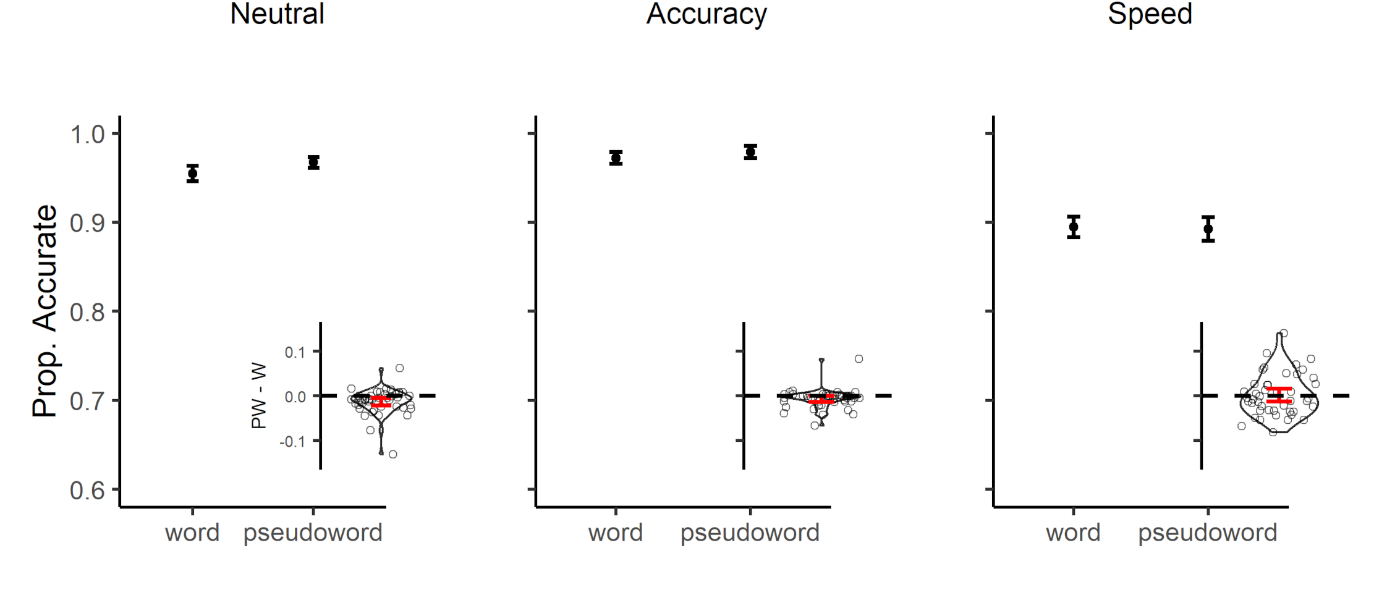


*Note*. Results for response accuracy as a function of SAT instructions (Neutral, first column; Accuracy, second column; Speed, third column). Points represent empirical means, with error bars highlighting 95% confidence intervals, adjusted for within-participants variables following Morey (2008). Lines represent models’ estimates. No lines appear when the contrast was not significant. Inset plots provide an overview of the lexicality effect across participants (PW = pseudoword; W = word). Points represent individual difference-scores between pseudowords and words in the corresponding measure, with the violin-plot describing their distribution. Red error-bars highlight 95% confidence-interval of the mean effect for the whole sample.

**Table S3**

*Parameters of the fixed effects for generalized LME model on CAFs.*

| Fixed Effects |  | Est | SE | z |
| --- | --- | --- | --- | --- |
| Intercept |  | 3.92 | 0.12 | 32.24 |
| SAT (acc.) |  | 0.69 | 0.08 | 8.83 |
| SAT (speed) |  | -1.09 | 0.06 | -18.30 |
| Lexicality (pseudo.) |  | 0.32 | 0.10 | 3.12 |
| Quantile, linear |  | 27.41 | 3.99 | 6.87 |
| Quantile, quadratic |  | -14.41 | 4.76 | -3.03 |
| SAT (acc) X Lexicality (pseudo.) |  | -0.16 | 0.12 | -1.37 |
| SAT (speed) X Lexicality (pseudo.) |  | -0.32 | 0.09 | -3.63 |
| SAT (acc.) X Quantile, linear |  | -26.58 | -26.58 | -3.91 |
| SAT (speed) X Quantile, linear |  | 16.14 | 5.09 | 3.17 |
| SAT (acc.) X Quantile, quadratic |  | 42.18 | 5.51 | 7.66 |
| SAT (speed) X Quantile, quadratic |  | 17.23 | 4.42 | 3.90 |
| Lexicality (pseudo.) X Quantile, linear |  | 0.05 | 4.61 | 0.01 |
| Lexicality (pseudo.) X Quantile, quadratic |  | -51.81 | 7.26 | -7.14 |
| SAT (acc) X Lexicality (pseudo.) X Quantile, linear |  | 39.64 | 6.96 | 5.70 |
| SAT (speed) X Lexicality (pseudo.) X Quantile, linear |  | 104.33 | 5.14 | 20.28 |
| SAT (acc) X Lexicality (pseudo.) X Quantile, quadratic |  | -28.53 | 8.12 | -3.51 |
| SAT (speed) X Lexicality (pseudo.) X Quantile, quadratic |  | 4.53 | 5.61 | 0.81 |

*Note***.** SE = standard error; pseudo. = pseudoword; acc. = accuracy.

**Table S4**

*Parameters of the fixed effects for generalized LME model on CIAFs.*

| Fixed Effects |  | Est | SE | z |
| --- | --- | --- | --- | --- |
| Intercept |  | -3.06 | 0.13 | 24.35 |
| SAT (acc.) |  | -0.70 | 0.06 | -11.73 |
| SAT (speed) |  | 0.87 | 0.05 | 18.10 |
| Lexicality (pseudo.) |  | -0.23 | 0.08 | -2.85 |
| Quantile, linear |  | -192.48 | 3.96 | -48.60 |
| Quantile, quadratic |  | 152.01 | 3.78 | 40.19 |
| SAT (acc) X Lexicality (pseudo.) |  | 0.11 | 0.09 | 1.26 |
| SAT (speed) X Lexicality (pseudo.) |  | 0.20 | 0.07 | 2.84 |
| SAT (acc.) X Quantile, linear |  | 15.39 | 5.66 | 2.72 |
| SAT (speed) X Quantile, linear |  | 14.73 | 7.42 | 1.99 |
| SAT (acc.) X Quantile, quadratic |  | -20.07 | 5.13 | -3.91 |
| SAT (speed) X Quantile, quadratic |  | -24.40 | 5.53 | -4.41 |
| Lexicality (pseudo.) X Quantile, linear |  | -7.64 | 5.15 | -1.48 |
| Lexicality (pseudo.) X Quantile, quadratic |  | 36.20 | 4.66 | 7.77 |
| SAT (acc) X Lexicality (pseudo.) X Quantile, linear |  | -2.88 | 5.34 | -0.54 |
| SAT (speed) X Lexicality (pseudo.) X Quantile, linear |  | -64.23 | 6.55 | -9.81 |
| SAT (acc) X Lexicality (pseudo.) X Quantile, quadratic |  | 3.95 | 5.02 | 0.79 |
| SAT (speed) X Lexicality (pseudo.) X Quantile, quadratic |  | -31.04 | 8.25 | -3.76 |

*Note***.** SE = standard error; pseudo. = pseudoword; acc. = accuracy.

**Figure S2**

*Results for measures of partial errors.*


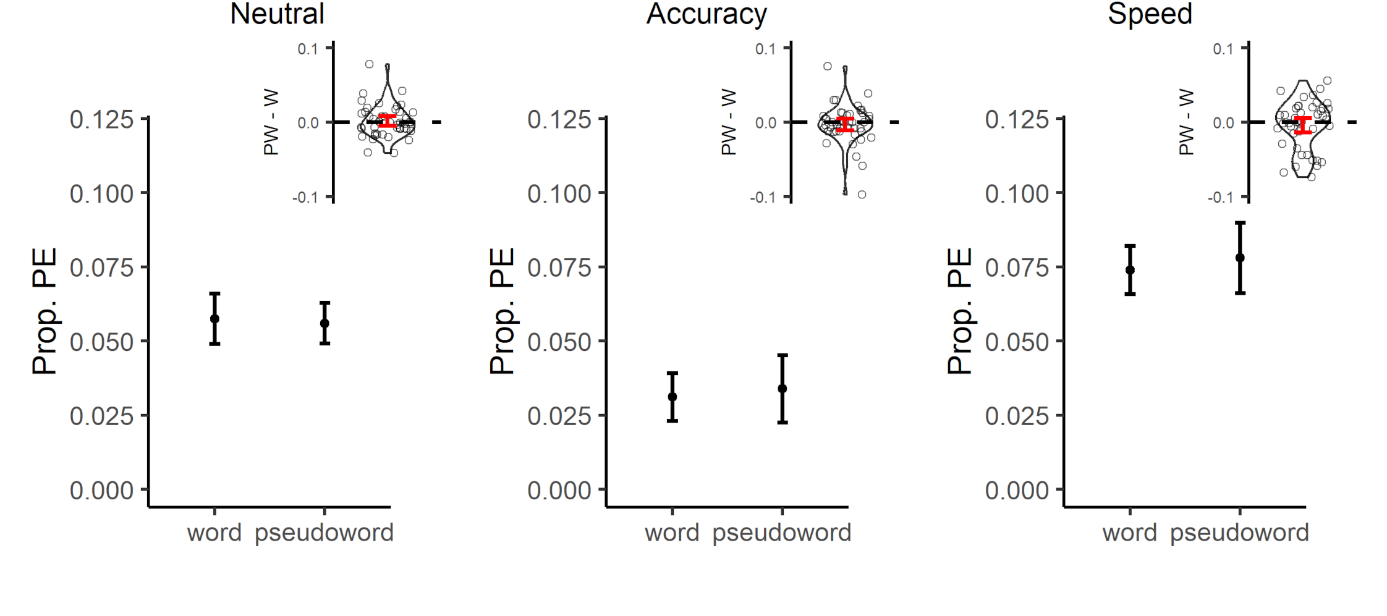


*Note*. Results for partial errors as a function of SAT instructions (Neutral, first column; Accuracy, second column; Speed, third column). Points represent empirical means, with error bars highlighting 95% confidence intervals, adjusted for within-participants variables following Morey (2008). No lines appear as the lexicality effect was not significant. Inset plots provide an overview of the lexicality effect across participants (PW = pseudoword; W = word). Points represent individual difference-scores between pseudowords and words in the corresponding measure, with the violin-plot describing their distribution. Red error-bars highlight 95% confidence-interval of the mean effect for the whole sample.
